# A beginner’s guide to the clinical approach and key concepts of glomerular disease: a mini review

**DOI:** 10.2478/abm-2025-0036

**Published:** 2025-12-31

**Authors:** Suwasin Udomkarnjananun, Talerngsak Kanjanabuch, Dennis A. Hesselink

**Affiliations:** Department of Medicine, Faculty of Medicine, Chulalongkorn University and King Chulalongkorn Memorial Hospital, The Thai Red Cross Society, Bangkok 10330, Thailand; Excellence Center for Organ Transplantation (ECOT), King Chulalongkorn Memorial Hospital, The Thai Red Cross Society, Bangkok 10330, Thailand; Center of Excellence in Renal Immunology and Renal Transplantation, Faculty of Medicine, Chulalongkorn University, Bangkok 10330, Thailand; Department of Nephrology and Transplantation, Erasmus MC, University Medical Center, Rotterdam 3015 GD, the Netherlands

**Keywords:** approach, diagnostic, glomerulonephritis, nephritis, nephrotic

## Abstract

Glomerular disease is a common issue in nephrology, where prompt diagnosis and appropriate treatment are crucial to halting disease progression and preventing the development of chronic kidney disease or end-stage kidney disease. The initial approach and differential diagnosis remain essential clinical skills for ensuring timely investigation and management. In an era of advanced molecular diagnostics in histopathology and biologic therapies, there is a notable gap in the literature regarding a general introduction to the overarching concepts of glomerular disease. This article aims to serve as a guide for medical students and internists, offering an overview of the approach to glomerular disease, including differential diagnoses, the clinical features of nephritis and nephrotic syndrome, and common presentations that aid in diagnosis, such as foamy urine, hematuria, and proteinuria.

Glomerular disease is a major entity in nephrology. Medical students, internists, and nephrology trainees often struggle with the differential diagnosis and clinical syndromes of glomerular disease. This difficulty can hinder their ability to pursue a deeper understanding of the field. While numerous articles provide comprehensive reviews of glomerular diseases, most focus on differential diagnosis through histopathology or advanced molecular testing [1–6]. However, there is a lack of resources emphasizing clinical presentation and basic investigations alongside a conceptual framework for differentiating these diseases.

This article aims to serve as a practical guide for medical students and internists, offering a practical approach to glomerular diseases. It provides an introduction to the differential diagnosis of nephritic versus nephrotic syndromes, emphasizing clinical features and the use of basic investigations to refine the likelihood of specific glomerular pathologies before kidney biopsy. As this review is intended as a beginner’s guide, detailed discussions on pathophysiology, histopathology, treatment, and prognosis of individual glomerular diseases will not be included. Readers seeking more comprehensive information on specific conditions are encouraged to refer to other excellent reviews [4–7].

## Spectrum of glomerular disease

Glomerular diseases are commonly classified into 2 main syndromes: nephritic syndrome and nephrotic syndrome [3, 4]. These 2 groups have distinct “classical” features that aid in identifying the underlying disease (Table 1).

**Table 1.** Classical features of nephritic and nephrotic syndrome

Nephritic syndrome	Nephrotic syndrome
Salt-water retention edema (increased hydrostatic pressure)	Loose part edema (decreased oncotic pressure edema)
• Starts in the dependent part (legs, ankles) Edema in other parts at a later stage	• Starts with loose tissue (upper eyelids, genitalia) Edema in dependent parts at a later stage
New onset or accelerated hypertension	Pleural effusion, ascites, hyperlipidemia, hypoalbuminemia
Dysmorphic RBC in UA	Usually, no RBC in UA, not dysmorphic if presented
RBC cast	Oval fat body (degenerating tubular epithelial cells filled that contain refractile fat droplets)
Nephrotic or sub-nephrotic range proteinuria	Nephrotic range proteinuria
Usually with elevated Cr/decreased eGFR	Meet all the criteria of nephrotic syndrome
	1. Generalized edema
	2. Nephrotic range proteinuria (≥3.5 g/d)
	3. Hypoalbuminemia (<3.0-3.5 g/dL, depends on assay)

1 Cr, creatinine; eGFR, estimated glomerular filtration rate; RBC, red blood cell; UA, urinalysis.

Patients with nephritic syndrome typically present with salt and fluid retention, leading to dependent edema (e.g., dorsum of the feet and pretibial edema) that may progress to generalized edema. This fluid retention often results in new-onset hypertension or worsening of preexisting hypertension. The primary mechanism of edema in nephritic syndrome is increased intravascular hydrostatic pressure, which results from a decreased glomerular filtration rate (GFR) and activation of the renin-angiotensin-aldosterone system. In contrast, edema in nephrotic syndrome is believed to arise from hypoalbuminemia and reduced oncotic pressure. It usually begins in areas with loose connective tissue, such as the eyelids, genitalia, or third spaces (e.g., pleural or peritoneal cavities), before spreading elsewhere. Notably, reduced oncotic pressure (i.e., the underfill hypothesis) does not fully explain edema in nephrotic syndrome. Instead, salt and water retention also significantly contributes to its development (the overfill hypothesis). One proposed mechanism involves activation of epithelial sodium channels in the cortical collecting ducts, triggered by plasmin as a component of proteinuria [8, 9]. In advanced stages, edema becomes more generalized in both conditions, making differentiation challenging. However, the sequence and pattern of edema can still guide initial impressions. A thorough history focusing on the site of initial edema can provide valuable insights for clinical diagnosis.

Nephrotic syndrome is often characterized by pleural effusion, ascites, hyperlipidemia (elevated cholesterol and triglycerides), hypoalbuminemia, nephrotic-range proteinuria, and the presence of oval fat bodies in the urine. In contrast, features more common in nephritic syndrome include dysmorphic red blood cells (RBCs) in the urine and RBC casts. Patients with nephritic syndrome are also more likely to be present with elevated serum creatinine or acute kidney injury (AKI), typically resulting from glomerular occlusion or crescent formation. Table 1 summarizes the classical characteristics of nephritic and nephrotic syndromes, although overlapping features can occur in some patients.

Foamy urine or increased bubbles in the urine are another potential indicator of proteinuria and glomerular disease. However, studies suggest that only 20%–30% of patients with foamy urine have significant proteinuria or albuminuria [10]. The foaming mechanism involves gas pockets trapped by amphiphilic surfactants, which can originate from proteins, amino acids, phospholipids (such as those in cell membranes), toilet cleaning chemicals, and certain urinary metabolites, including bile salts or fatty acid esters [11]. While foamy urine is often associated with proteinuria, it can also result from increased urinary metabolites with amphiphilic properties after meals. When assessing proteinuria, the type of protein is an important consideration. Dipstick tests detect only albuminuria, which is sufficient in most cases since 30%–60% of protein in glomerular diseases consists of albumin [12]. However, in certain conditions, such as multiple myeloma with cast nephropathy, the predominant proteins in urine are immunoglobulin (Ig) light chains rather than albumin, as the pathology is non-glomerular. In these cases, dipstick tests may yield a negative result for albuminuria (proteinuria-albuminuria discrepancy), potentially reducing suspicion of myeloma kidney if proteinuria is not specifically evaluated [13].

Dysmorphic RBC in the urine of nephritic patients result from multiple processes, including the passage ofRBCs through the glomerular basement membrane (GBM) and osmotic injury during transit through hypotonic tubular segments. The average RBC diameter is 6–8 μm, whereas the endothelial fenestration diameter is only 0.06–0.08 μm [14]. Increased glomerular hydrostatic pressure and impaired GBM integrity in glomerulonephritis (GN) compromise the RBC membrane during diapedesis, leading to poikilocytosis. Subsequent exposure to the hypotonic environment in the distal tubule contributes to hypochromia and anisocytosis in dysmorphic RBC [14–17]. Dilute urine, such as in patients receiving diuretics, can selectively lyse dysmorphic RBCs, leaving only normal RBCs and potentially reducing the sensitivity of GN diagnosis [15, 16]. In clinical practice, the sensitivity and specificity of dysmorphic RBCs for diagnosing glomerular disease depend on the cutoff values used. For instance, a dysmorphic RBC proportion ≥20% provides 93.0% sensitivity and 34.0% specificity for GN, while a cutoff of ≥50% increases specificity to 43.0% with comparable sensitivity [18]. Although dysmorphic RBCs can present with various morphological features (e.g., stomatocytes, schizocytes, codocytes, and echinocytes), acanthocytes (ring-shaped cells with vesicle-like protrusions) are the most characteristic of glomerular bleeding. Acanthocyturia ≥5% shows a sensitivity of 28.0%–40.0% and specificity of 95.0%–97.0% for GN [18–20]. The optimal method for evaluating dysmorphic RBCs is phase-contrast light microscopy [14]. However, 1 study found that using standard light microscopy with the condenser lens lowered was equally effective for guiding hematuria evaluation [21].

Microscopic hematuria is relatively uncommon in patients with nephrotic syndrome, and dysmorphic RBCs are even rarer compared to those seen in nephritis. One study reported that at least 60.0% of patients with nephritis—such as pauci-immune GN, IgA nephropathy (IgAN), and lupus nephritis (LN)—had RBCs in their urine, with 20.0%–30.0% showing dysmorphic RBCs at proportions ≥25%. Interestingly, the same study found that 50.0% of patients with membranous nephropathy (MN), typically classified as nephrotic syndrome, had microscopic hematuria, and 20.0% of the overall patients exhibited dysmorphic RBCs at proportions of ≥25% [22]. However, it was not clearly specified whether these MN cases included LN class V.

AKI or abnormal serum creatinine levels can occur in patients with nephrotic syndrome but are generally less severe than in those with nephritis. The primary mechanism of AKI in nephrotic syndrome is decreased intravascular volume due to plasma leakage from hypoalbuminemia, leading to prerenal azotemia. Other potential mechanisms include renal vein thrombosis, resulting from urinary loss of anticoagulant proteins, and concurrent interstitial or tubular inflammation, as observed in cases of non-steroidal anti-inflammatory drug (NSAID)-induced nephritis.

Dyslipidemia is common in nephrotic syndrome, although it is not required for diagnosis. Lipid abnormalities typically include elevated levels of cholesterol, triglycerides, low-density lipoprotein (LDL), intermediate-density lipoprotein (IDL), very low-density lipoprotein (VLDL), and lipoprotein(a) [23]. The activity of lipoprotein lipase (LPL) and hepatic lipase is reduced in nephrotic syndrome, contributing to increased levels of LDL, IDL, and VLDL, partly due to the loss of LPL activators [24]. However, impaired clearance alone does not fully explain the dyslipidemia; increased synthesis also plays a significant role. For instance, acetyl-CoA carboxylase and fatty acid synthase levels are elevated, as evidenced by increased hepatic gene expression in nephrotic rats [25]. These lipid abnormalities can lead to nephrotoxicity through inflammation and damage to glomerular endothelial and tubular epithelial cells [24]. Additionally, plasma levels of proprotein convertase subtilisin/kexin type 9 (PCSK9) are elevated due to podocyte damage, making PCSK9 inhibitors a potential therapeutic target in nephrotic syndrome [26]. High-density lipoprotein (HDL) levels in nephrotic patients can vary, but the HDL cholesterol-to-total cholesterol ratio is typically reduced [27].

The overall prevalence of glomerular diseases varies significantly across regions and ethnicities. Table 2 highlights the prevalence of kidney biopsy pathological diagnoses in different regions, including the USA/Canada, Europe, Latin America, Japan, and Thailand [28–30].

**Table 2.** Prevalence of kidney biopsy pathological diagnosis (top 10 most common) [28–30]

USA/Canada (n = 23,391) Survey 2012–2013	Europe (n = 15,042) Survey 2012–2013	Latin America (n = 2,561) Survey 2012–2013	Japan (n = 28,728) 2007–2017	Thailand (n = 5,893) 2000–2014
FSGS (19%)	IgAN/HSP (22%)	LN (38%)	IgAN/HSP (33%)	LN (32%)
DN (19%)	FSGS (15%)	FSGS (16%)	MN (9%)	IgAN/HSP (21%)
IgAN/HSP (12%)	MN (13%)	MN (11%)	FSGS/NS (9%)	MCD (8%)
MN (12%)	LN (10%)	MCD (7%)	DN (6%)	DN (8%)
LN (10%)	Pauci-immune GN (8%)	IgAN/HSP (6%)	Pauci-immune GN (6%)	FSGS (7%)
Pauci-immune GN (5%)	DN (7%)	Pauci-immune GN (5%)	MCD (6%)	MN (7%)
MCD (4%)	MCD (6%)	DN (4%)	LN (5%)	TI disease (4%)
Alport syndrome/TBM (3%)	Amyloidosis (4%)	MPGN (3%)	TI disease (4%)	Infection-related GN (3%)
TMA (3%)	MPGN (4%)	MesProlif GN (2%)	Amyloidosis (2%)	Pauci-immune GN (3%)
MPGN (3%)	TMA (2%)	Infection-related GN (2%)	Alport syndrome/TBM (1%)	MPGN (2%)

1 DN, diabetic nephropathy; FSGS, focal segmental glomerulosclerosis; GN, glomerulonephritis; HSP, Henoch-Schönlein purpura; IgAN; IgA nephropathy; LN, lupus nephritis; MCD, minimal change disease; MesProlif, mesangial proliferative; MN, membranous nephropathy; MPGN, membranoproliferative glomerulonephritis; NS, nephrosclerosis; TBM, thin basement membrane disease; TI, tubulointerstitial; TMA, thrombotic microangiopathy.

## Overview of nephritic syndrome

**Figure 1.**
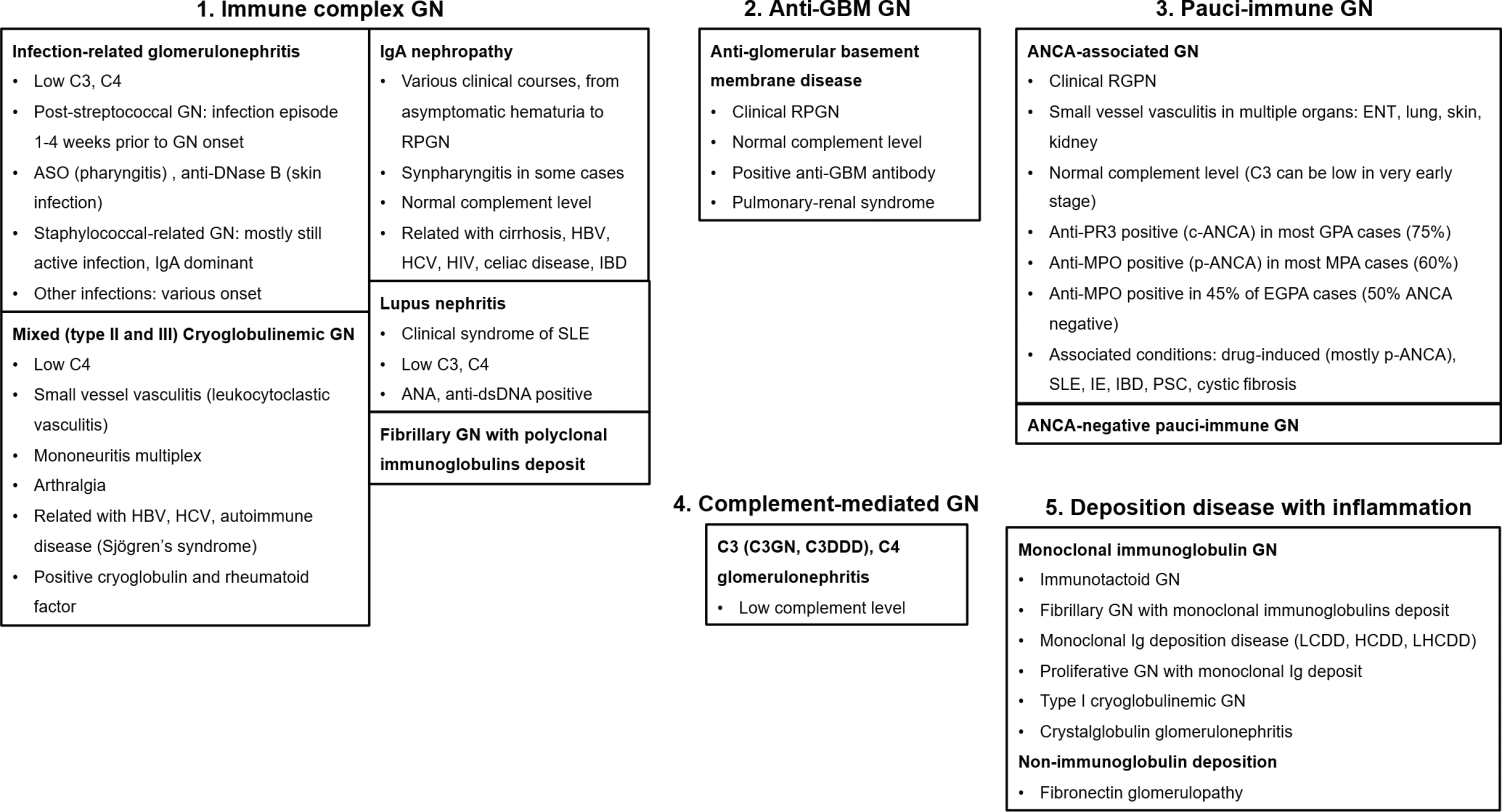
Overview of nephritic syndrome, listing common causes of GN and their classical presentations. ANA, anti-nuclear antibody; ANCA, anti-neutrophil cytoplasmic antibodies; ASO, anti-streptolysin-O; DDD, dense deposition disease; DN, diabetic nephropathy; EGPA, eosinophilic granulomatosis polyangiitis; ENT, ear-nose-throat; FSGS, focal segmental glomerulosclerosis; GBM, glomerular basement membrane; GN, glomerulonephritis; GPA, granulomatosis polyangiitis; HBV, hepatitis B virus; HCDD; heavy chain deposition disease; HCV, hepatitis C virus; HIV, human immunodeficiency virus; HSP, Henoch-Schönlein purpura; IBD, inflammatory bowel disease; IE, infective endocarditis; Ig, immunoglobulin; IgAN, IgA nephropathy; LCDD, light chain deposition disease; LHCDD, light-heavy chain deposition disease; MCD, minimal change disease; MesProlif, mesangial proliferative; MN, membranous nephropathy; MPA, microscopic polyangiitis; MPGN, membranoproliferative glomerulonephritis; MPO, myeloperoxidase; NS, nephrosclerosis; PR3, proteinase-3; PSC, primary sclerosing cholangitis; RPGN, rapidly progressive glomerulonephritis; SLE, systemic lupus erythematosus; TBM, thin basement membrane disease; TI, tubulointerstitial; TMA, thrombotic microangiopathy.

Nephritic syndrome, or GN, can be categorized into 5 primary types based on pathophysiology. Figure 1 provides an overview of these types: (1) immune complex-mediated GN, (2) anti-GBM disease, (3) pauci-immune GN, (4) complement-mediated GN, and (5) inflamed deposition disease particularly monoclonal Ig-associated GN. This classification serves to enhance familiarity with nephritic diseases and is not intended to provide an exhaustive discussion of each. Although distinct features define these types, overlapping features can occur in atypical cases.

Before performing a kidney biopsy, evaluating complement levels (commonly C3 and C4) can provide essential diagnostic clues [31–33]. As depicted in Figure 1, nephritic syndromes with hypocomplementemia include immune complex-mediated and complement-mediated GN. In contrast, complement levels in anti-GBM disease, pauci-immune GN, and monoclonal Ig-associated GN are typically normal. Some overlap between groups can occur. For instance, monoclonal Ig-associated GN may present with hypocomplementemia and pathological features resembling C3 GN, as monoclonal antibodies can disrupt the alternative complement pathway [34]. In such cases, additional diagnostic steps, such as pronase treatment of paraffin-embedded tissue, may be necessary to identify monoclonal deposits in the glomeruli [35]. This scenario is particularly pertinent in patients >50 years old who present with features suggestive of C3 glomerulopathy [36].

Table 3 summarizes complement levels across different GN types. It is important to recognize that non-primary glomerular conditions, such as atheroembolic disease, can also result in hypocomplementemia. Likewise, systemic conditions like sepsis, malnutrition, or hepatic failure may lead to reduced complement levels even in the absence of glomerular disease. Although IgAN is an immune complex-mediated condition, complement levels are typically normal. The activation of the alternative and lectin complement pathways plays a key role in its pathogenesis. The exact mechanism underlying normocomplementemia in IgAN is not fully understood. Proposed mechanisms include localized complement activation in the glomeruli, as opposed to the systemic activation observed in other immune complex diseases [37, 38] and IgA’s weaker ability to fix complement compared to IgG or IgM [39]. While uncommon, low C3 levels in patients with IgAN have been associated with a poorer prognosis [38], as well as a high serum IgA-to-C3 ratio [40]. Another immune complex disease, LN, encompasses multiple classes and often presents with hypocomplementemia, particularly in class III and IV (proliferative classes), which occur in 70%–80% of cases. In contrast, class V LN (membranous type) is less likely to exhibit hypocomplementemia, with rates of 30%–50%) [41, 42].

**Table 3.** Complement levels in GN with classical and alternative pathway

Pathway	Disease	C3	C4
Classical	LN	↓	↓↓
pathway	Mixed cryoglobulinemic GN	↓ or ↔	↓↓
Alternative	C3 GN	↓ or ↔	↔
pathway	Infection-related GN	↓	↓ or ↔
	Atypical hemolytic-uremic syndrome	↓	↔
	Atheroembolic disease	↓ or ↔	↓ or ↔
Other non-GN conditions that cause hypocomplementemia			
• Severe sepsis			
• Malnutrition			
• Hepatic failure			

1 GN, glomerulonephritis; LN, lupus nephritis.

From physical examination, one of the signs that can be detected during the physical examination is cutaneous vasculitis, which can accompany certain glomerular diseases. These skin lesions typically present as palpable purpura or leukocytoclastic vasculitis, characterized by neutrophilic infiltration within and around the vessel wall, accompanied by fibrinoid necrosis and damage to the surrounding tissue [43]. Glomerular diseases associated with these small vessel vasculitides include antineutrophil cytoplasmic antibodies (ANCA)-associated GN, IgA vasculitis (Henoch-Schönlein purpura), cryoglobulinemic vasculitis, and systemic lupus erythematosus [44]. Clinicians can use these additional diagnostic clues to help narrow down the differential diagnosis and prompt further investigations.

**Figure 2.**
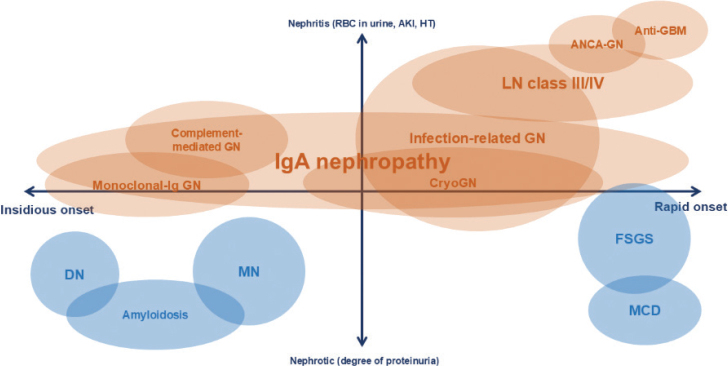
Overview of glomerular diseases according to their typical onset and clinical phenotypes. Brown represents diseases with predominantly nephritic features, while blue represents diseases with predominantly nephrotic features. (Adapted with permission from Dr. Ali Poyan Mehr) [45]. AKI, acute kidney injury; DN, diabetic nephropathy; FSGS, focal segmental glomerulosclerosis; GBM, glomerular basement membrane; GN, glomerulonephritis; MCD, minimal change disease; MN, membranous nephropathy; RBC, red blood cell.

The details of each GN, beyond the key features listed in Figure 1, are beyond the scope of this review, which serves as an introduction to the concept of glomerular disease. However, to provide a clearer understanding of the disease’s natural history, Figure 2 illustrates the clinical phenotypes and onset of nephritis (and nephrotic) syndrome [45]. Some nephritic syndromes almost always present as acute GN or rapidly progressive glomerulonephritis (RPGN), characterized by a rapid decline in kidney function over days to weeks, often accompanied by new-onset or accelerated hypertension. These include ANCA-associated GN and anti-GBM disease. Notably, these 2 conditions typically lack significant proteinuria, such as nephrotic-range proteinuria, which is more commonly observed in immune complex-mediated diseases or monoclonal Ig-associated GN. Monoclonal Ig-associated GN usually has a more insidious onset, manifesting as chronic GN with a gradual decline in kidney function over several months. IgAN displays the broadest spectrum of clinical manifestations, ranging from asymptomatic hematuria, chronic GN, acute nephritis, or RPGN. Please note that Figure 2 presents only the usual or classic clinical presentations and does not aim to cover all aspects of each disease’s clinical manifestations. In addition, congenital nephritis syndromes, particularly Alport syndrome and its variants (caused by collagen type 4 *(COL4) A3, COL4A4*, and *COL4A5* mutations; previously referred to as thin basement membrane disease), should be considered in young patients presenting with hematuria (microscopic or gross), proteinuria, and extra-renal features, such as sensorineural hearing loss, with or without a family history of kidney disease [46].

GN can be mimicked by several conditions, such as acute tubular injury or interstitial nephritis, which present with AKI. However, these conditions should be excluded based on urine analysis and the specific clinical features of GN. One systemic condition that may mimic nephritis is thrombotic microangiopathy (TMA)-related vasculopathy [47]. Examples include atypical hemolytic uremic syndrome, anti-phospholipid syndrome, scleroderma renal crisis, medication-induced TMA, or malignant hypertension from various causes, all of which present severe or accelerated hypertension and deteriorating kidney function [47, 48]. These small-vessel vasculopathies often manifest as a GN-like syndrome (AKI with RBCs in the urine, usually non-dysmorphic); however, a thorough evaluation of systemic clues and a peripheral blood smear can aid in the differential diagnosis. Additionally, preeclampsia or hemolysis, elevated liver enzymes, and low platelet syndrome—a vasculopathy that pathologically demonstrates glomerular endotheliosis—should also be considered in the differential diagnosis for pregnant patients with suspected glomerular disease [49].

## Overview of nephrotic syndrome

The cardinal features of nephrotic syndrome include nephrotic-range proteinuria, hypoalbuminemia, and generalized edema (Table 1). Each disease can be categorized by its pathophysiology, and the onset of disease often provides valuable diagnostic clues.

Podocytopathies are diseases in which podocytes are directly affected, including minimal change disease (MCD) and focal segmental glomerulosclerosis (FSGS). These conditions typically present with a rapid onset of edema or foamy urine, leading to a hospital visit within days to weeks. In contrast, MN has a more insidious onset, which can be explained by the slower immune complex formation in its pathophysiology. Deposition diseases and diabetic nephropathy (DN) tend to have a longer time from onset to hospital visit, usually due to their slow progression, ranging from months to even years. Figure 2 illustrates the spectrum of nephrotic (and nephritic) syndromes, highlighting the different onset times for various diseases.

**Figure 3.**
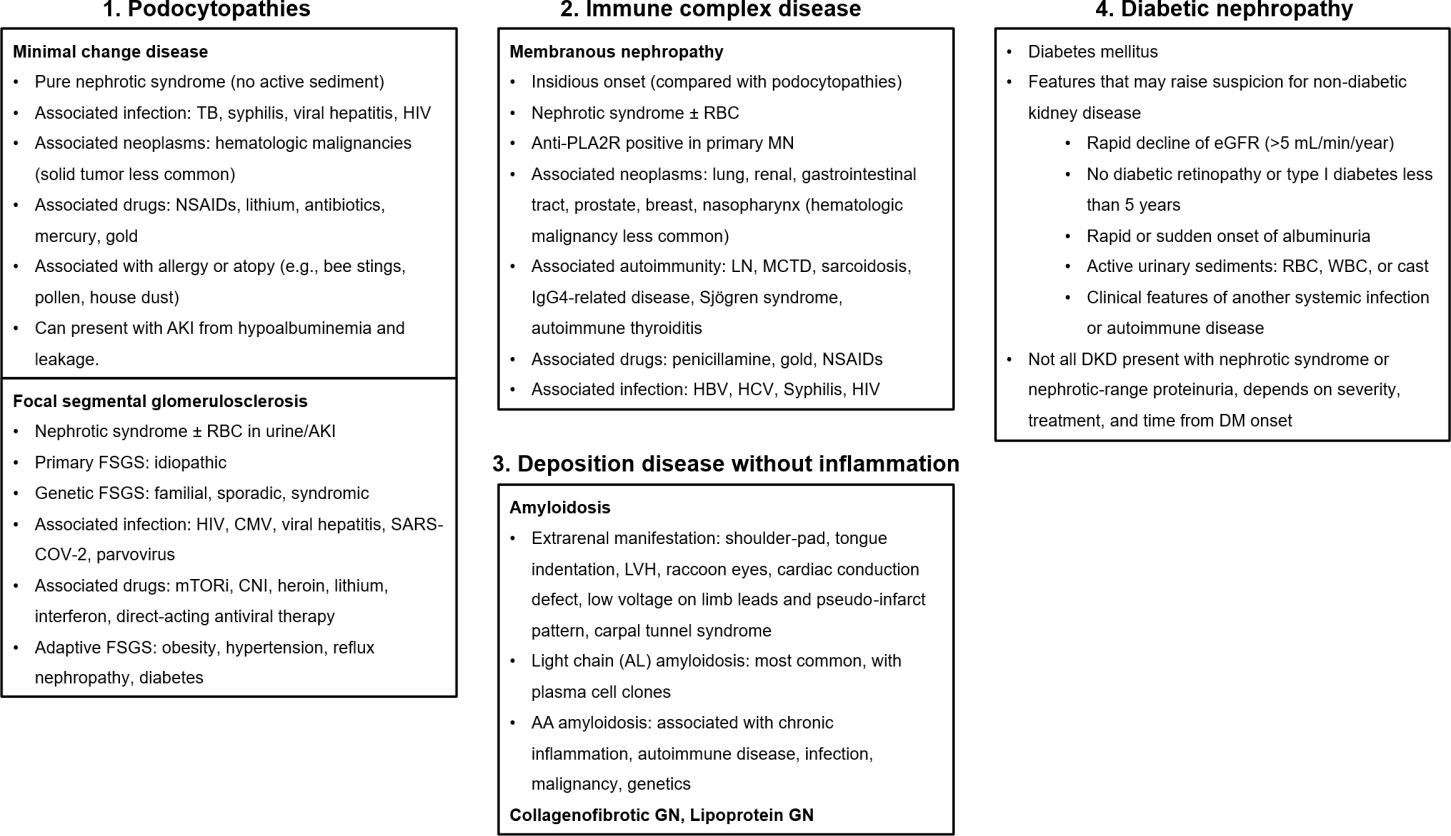
Overview of nephrotic syndrome, listing common causes of GN and their classical presentations. AKI, acute kidney injury; CMV, cytomegalovirus; CNI, calcineurin inhibitor; DKD, diabetic kidney disease; eGFR, estimated glomerular filtration rate; FSGS, focal segmental glomerulosclerosis; GN, glomerulonephritis; HBV, hepatitis B virus; HCV, hepatitis C virus; HIV, human immunodeficiency virus; LN, lupus nephritis; LVH, left ventricular hypertrophy; MCTD, mixed connective tissue disease; MN, membranous nephropathy; mTORi, mammalian target of rapamycin inhibitor; NSAID, non-steroidal anti-inflammatory drug; PLA2R, phospholipase A2 receptor; RBC, red blood cell; SARS-COV-2, severe acute respiratory syndrome coronavirus 2; WBC, white blood cell.

The overview of each distinct nephrotic syndrome is illustrated in Figure 3. A few key points should be noted. First, the list highlights the typical or classical presentations of these diseases and does not encompass all possible manifestations. Second, particular attention should be given to the clinical heterogeneity of FSGS. FSGS can be classified etiologically into primary (formerly termed “idiopathic”) FSGS, genetic FSGS, secondary FSGS (caused by viral infections, drug use, or adaptive changes), and FSGS of undetermined cause (FSGS-UC) [7]. Unlike other forms of FSGS, adaptive (or “secondary”) FSGS (commonly linked to chronic hypertension or obesity) and FSGS-UC often do not present with overt nephrotic syndrome. Instead, these patients typically exhibit nephrotic-range or subnephrotic-range proteinuria, borderline hypoalbuminemia or normal serum albumin levels, and mild or absent pedal edema [3, 7]. Pathologically, FSGS is further classified into 5 morphological variants per the Columbia classification: collapsing, tip, cellular, perihilar, and not otherwise specified (NOS) [50]. Adaptive FSGS is usually observed as the perihilar or NOS variant on biopsy, often displaying significantly fewer clinical features of full-blown nephrotic syndrome [51]. Unlike other variants, which indicate direct podocyte injury, adaptive FSGS and perihilar/NOS variants are associated with more chronic processes, often secondary to systemic conditions that contribute to chronic kidney disease (CKD). Clinicians should recognize that FSGS is a heterogeneous entity, with variations in its clinical, pathological, and etiological presentations.

DN is a well-defined pathological condition primarily affecting the glomeruli, characterized by a progressive process with 4 stages, in which albuminuria is a key feature. In contrast, diabetic kidney disease (DKD) is a broader term that refers to CKD in patients with diabetes mellitus, encompassing damage to not only the glomeruli but also the renal vasculature and tubulointerstitial structures [52, 53]. The diagnosis of DKD is typically based on clinical criteria, such as a decline in GFR and/or the presence of albuminuria, without the need for a kidney biopsy. Notably, non-albuminuric DKD is an emerging entity with a generally better prognosis compared to albuminuric DKD [54, 55]. It is crucial to recognize that not all patients diagnosed with DKD manifest with nephrotic syndrome or exhibit nephrotic-range proteinuria. The presence and extent of proteinuria are generally influenced by the stage of disease at presentation, the duration of diabetes mellitus, and the therapeutic interventions administered. Advances in DKD treatment—including renin-angiotensin system inhibitors, sodium-glucose cotransporter 2 inhibitors, non-steroidal mineralocorticoid antagonists, and glucagon-like peptide-1 receptor agonists—have significantly reduced progression to full-blown nephrotic syndrome in patients receiving timely and adequate treatment. However, it is crucial to recognize that not all diabetes mellitus patients with proteinuria or albuminuria have DKD. Certain red flags should prompt consideration of alternative diagnoses, including rapid decline in GFR (>5 mL/min/year), absence of diabetic retinopathy, type 1 diabetes mellitus duration of <5 years, sudden or rapid onset of albuminuria, active urinary sediment (e.g., dysmorphic RBCs, white blood cells, or casts), and clinical signs of other systemic autoimmune diseases [56, 57]. In such cases, a kidney biopsy is warranted to exclude non-DKDs.

## Summary and considerations for differential diagnosis

In summary, when patients present with foamy urine, generalized edema, or other symptoms suggestive of glomerular disease, the initial step is to confirm whether they have a glomerular disorder and determine if it is nephritic or nephrotic syndrome. Differential diagnoses should also include other causes of AKI, such as acute interstitial nephritis, acute tubular injury, or small vessel thrombosis (e.g., TMA). These figures outline the typical presentations of nephritic and nephrotic syndromes, which are primarily associated with immune-mediated diseases. However, clinicians should also consider indirect immune-mediated glomerular diseases, such as adaptive FSGS secondary to chronic hypertension or DN, particularly when the clinical presentation does not align fully with nephritic or nephrotic syndrome in proteinuric patients.
